# Implementation of the FAST HUG if WEAK extended ward round checklist in a tertiary interdisciplinary intensive care unit: a before-and-after quality improvement study

**DOI:** 10.62675/2965-2774.20260158

**Published:** 2026-02-20

**Authors:** Raphael Klar, Alexander Hoffmann, Jasmin Blumer, Luca Cioccari

**Affiliations:** 1 Kantonsspital Aarau Department of Intensive Care Medicine Aarau Switzerland Department of Intensive Care Medicine, Kantonsspital Aarau - Aarau, Switzerland.

Checklists have become an integral component of healthcare quality improvement, with clear evidence of benefits for patient outcomes.^(1)^ In intensive care units (ICUs), structured bedside ward-round checklists are linked to reductions in length of stay, duration of mechanical ventilation, ventilator-associated pneumonia, and ICU mortality.^(2-4)^ However, their routine implementation in clinical practice remains inconsistent, often due to design flaws, low acceptance, workload concerns, and cultural resistance.^(5-8)^ In this exploratory implementation study, we evaluated whether implementing an extended ward-round checklist, integrated into our electronic health record (EHR) system, could improve ward-round structure and reduce perceived prescription errors.

This study was conducted in a 24-bed interdisciplinary ICU at a tertiary care hospital in Switzerland. Prior to this study, daily ward rounds were led by ICU staff specialists with the participation of ICU registrars and nurses. Pharmacists, physiotherapists, and other allied health staff were not routinely included, and no structured checklists were used. To identify frequently overlooked tasks and areas for improvement in ward-round structure, we distributed a baseline survey to all ICU staff via e-mail on May 5, 2022. A reminder followed after 7 days, and the survey closed on May 16. Participation was voluntary and anonymous; ethics approval was not required under Swiss regulations (Human Research Act, Article 2). Based on a targeted literature review and survey findings, we developed an extended checklist that included items such as weaning, ICU discharge planning, antibiotic management, and catheter removal. We intentionally adopted the "FAST HUG" acronym, an established critical care mnemonic originally proposed by Vincent et al.^(9)^ To adapt it to our setting, we combined elements from the original version with survey feedback and expert input, resulting in a tailored tool ("FAST HUG if WEAK") that reflects both evidence-based practice and local needs. The checklist was integrated into the EHR on May 16, 2022, and a multi-modal implementation strategy supported its rollout. Staff were informed via e-mail, a departmental newsletter, and interprofessional training sessions, which were video-recorded and made permanently available. A clinical nurse specialist was available on the unit for ongoing questions and actively promoted checklist use during day shifts. To support sustained adoption, we also created a concise visual summary highlighting the checklist's core elements and rounding flow. A follow-up survey was distributed on July 18, 2022, to assess the following components of the RE-AIM model:^(10)^ *R*each (response rate), *E*ffectiveness (perceived impact on ward-round structure and continuity, changes in prescription error frequency), *A*doption (checklist use), *I*mplementation barriers (relevance, user-friendliness), and potential for *M*aintenance (ongoing use). The full list of survey questions is provided in the Appendix. A reminder was sent after 11 days, and the survey closed on August 10, 2022. Data were analysed descriptively. If appropriate, Fisher's exact test was applied to compare selected response items between groups. As the analyses were exploratory and not based on predefined hypotheses for each comparison, we did not apply multiple-testing corrections.

Of the 175 ICU staff invited, 123 responded to the baseline survey (response rate 70.3%; 105 nurses, 18 physicians). Frequently overlooked tasks prior to checklist development included updating do-not-resuscitate plans, medication reviews, catheter removal, and the assessment of analgesia and sedation. Most respondents (86% of nurses and 67% of physicians) supported improving the structure of ward rounds, highlighting continuity, communication, and accuracy of orders as key priorities.

Following the checklist implementation, 111 of 185 staff (60%) completed the follow-up survey. Most reported improved ward-round structure and fewer prescription errors (Table 1). Key domains of critical care, such as nutrition, sedation, analgesia, thromboprophylaxis, and end-of-life care, were less frequently missed. Nurses more often reported better attention to nutrition (62% *versus* 30%; p = 0.02), sedation (36% *versus* 0%; p < 0.001), analgesia (24% *versus* 0%; p = 0.01), and thromboprophylaxis (35% *versus* 10%; p = 0.02). In comparison, physicians noted improvements in catheter removal (65% *versus* 16%; p < 0.001), stress ulcer prophylaxis (55% *versus* 22%; p = 0.01), and glucose management (45% *versus* 9%; p < 0.001). Checklist use among physicians was high, with 65% reporting daily use and 35% reporting using it multiple times per week. Nearly all physicians (95%) considered the checklist both appropriately concise and helpful. Continued use was supported by 97% of nurses and 95% of physicians.

**Table 1:** Intensive care unit staff's perception of prescription errors and ward round structure before and after checklist implementation

Question			Yes	Rather yes	Rather no	No
Before implementation						
	High number of prescription errors					
		Nurses	32 (26)	49 (40)	39 (32)	1 (1)
		Doctors	0	6 (33)	12 (67)	0
	Need for improvement of ward rounds*					
		Nurses and doctors	116 (83)			24 (17)
After implementation						
	Reduced the number of prescription errors					
		Nurses	8 (7)	70 (63)	25 (22)	9 (8)
		Doctors	9 (16)	30 (53)	17 (31)	0
	Improvement of ward rounds					
		Nurses	26 (23)	67 (59)	16 (14)	5 (4)
		Doctors	11 (20)	30 (55)	14 (25)	0
	Supports continued checklist use*					
		Nurses	108 (97)			3 (3)
		Doctors	105 (95)			6 (5)

* Only yes/no options were provided for this item. Results expressed as n (%).

A key strength of this study was its multimodal implementation strategy and structured baseline needs assessment, which systematically identified common omissions during ward rounds and guided the design of the extended checklist. However, the study relied entirely on subjective perceptions and lacked objective clinical endpoints, limiting the strength of its conclusions. Voluntary, anonymous surveys may have introduced response bias, as those with extreme views may have been more likely to respond. Checklist use was also self-reported without direct monitoring. As a single-centre study in a tertiary ICU with an EHR system, generalizability to lower-resource settings may be limited. However, the checklist could be adapted for use with paper forms or bedside posters. While early acceptance was strong and checklist use has continued beyond the study period, long-term sustainability remains to be formally assessed. Implementation challenges included perceived time constraints during rounds, concerns about additional documentation, and differing views on the utility of checklists. These were addressed through targeted training, visible clinical leadership, peer support, and ongoing feedback. The follow-up survey showed that the checklist content was perceived as highly relevant, user-friendliness was rated fairly good, and almost all physicians (95%) considered the checklist's scope appropriate.

In conclusion, we implemented an extended ICU ward-round checklist, resulting in promising improvements in perceived ward-round structure and prescription errors. Framed within the RE-AIM model (Reach, Effectiveness, Adoption, Implementation, and Maintenance),^(10)^ the intervention demonstrated substantial reach, with response rates of 70.3% at baseline and 60.0% at follow-up. Effectiveness was reflected in improved attention to key domains, including nutrition, sedation, catheter removal, and glucose control. Self-reported adoption was high: 65% of physicians used the checklist daily, and over 95% of staff supported continued use. Implementation was facilitated through interprofessional training, EHR integration, real-time feedback, and accessible learning tools, and maintenance has been sustained beyond the study period to the present day. While preliminary and exploratory, these findings suggest the checklist's acceptability and its potential for broader implementation in ICU and acute care settings.

## Data Availability

The contents underlying the research text are included in the manuscript. Original data will be made available on demand to the corresponding author upon reasonable request.

## Appendix

The survey instruments were developed specifically for this quality improvement project, based on a targeted literature review and expert consensus among intensive care unit (ICU) staff and clinical leads. The surveys were pre-tested for face validity and clarity among a small group of ICU staff (n=5) not involved in the project team, and minor adjustments were made accordingly.

**Table:**

**A. Baseline survey** Which of the following best describes your profession? - ICU nurse - ICU physician - Other (please specify) Which aspects do you feel are most often forgotten or overlooked during daily ICU ward rounds? - End-of-life care (e.g., updated do-not-resuscitate orders [DNR], advance directives) - Review of medications - Discussion about removing unneeded catheters - Adequate analgesia - Optimization of sedation - Ulcer prophylaxis - Glucose management - Weaning from mechanical ventilation - Nutritional strategy - Deep venous thrombosis [DVT] prophylaxis/anticoagulation Do you believe there is a need to improve the structure or continuity of the daily ICU ward round? - Yes - No - Unsure In your opinion, are there many prescription errors (i.e., medication orders) in the ICU? - Yes - Likely yes - Likely no - No
**B. Follow-up survey** Which of the following best describes your profession? - ICU Nurse - ICU Physician - Other (please specify) **Questions for nurses** Have you noticed an improvement in the structure and continuity of ward rounds since the checklist was introduced? - Yes - No - Unsure From your perspective, have prescription errors decreased since the introduction of the checklist? - Yes - No - Unsure Which aspects are now less frequently forgotten or overlooked on rounds? - Nutritional strategy - Review of medications - Optimization of sedation - DVT prophylaxis/anticoagulation - End-of-life care (updated DNR orders, etc.) - Adequate analgesia - Ulcer prophylaxis - Discussion about removing unneeded catheters - Glucose management - Weaning from mechanical ventilation - None of the above Do you think the use of this checklist should continue? - Yes - No - Unsure
**Questions for physicians** 2. How often have you used the checklist during your daily patient rounds? - Daily - Several times per week - Rarely - Never 3. On a scale of 1 (poor) to 5 (excellent), how do you rate the following aspects of the checklist? - Clarity of content - Relevance of included items - Design/layout - User-friendliness 4. Do you find the scope (length/number of items) of the checklist appropriate? - Yes - No (too long) - No (too short) 5. Overall, do you find the checklist helpful in ensuring that important aspects of care are not forgotten? - Yes - No - Unsure 6. Have you noticed an improvement in ward-round structure since the introduction of the checklist? - Yes - No - Unsure 7. From your perspective, have prescription errors decreased since the introduction of the checklist? - Yes - No - Unsure 8. Which aspects are now less frequently forgotten or overlooked on rounds? - Nutritional strategy - Review of medications - Optimization of sedation - DVT prophylaxis/anticoagulation - End-of-life care (resuscitation status, etc.) - Adequate analgesia - Ulcer prophylaxis - Discussion about removing unneeded catheters - Glucose management - Weaning from mechanical ventilation - None of the above 9. Do you think the use of this checklist should continue? - Yes - No - Unsure
**C. Final version of the extended checklist** *Mnemonic: "Give the patient a FAST HUG if he/she is WEAK!"* **Feeding and fluids** - Is the patient's nutritional strategy (enteral, parenteral) defined and optimized (calorimetry)? - Is the patient tolerating the feeding (diarrhoea, constipation, gastric residual volumes)? - Have fluid input and fluid balance been checked and adapted? **Analgesia** - Does the patient have adequate pain control? Can opioids be weaned? **Sedation** - Is sedation level appropriate? Can sedation be reduced? - Has an "awake and breathing" trial been performed? Is the patient waking? - Are there signs of neurological impairment or delirium? **Thromboprophylaxis** - Is it contraindicated? - If not, is the patient on heparin or Low molecular weight heparin or sequential compression devices? **Head-of-bed elevation** - Is the head of the bed elevated by at least 30° if on mechanical ventilation or at high risk of aspiration? **Ulcer prophylaxis** - Is stress ulcer prophylaxis indicated? **Glucose management** - Is the patient's glucose level adequately controlled? **Weaning** - If mechanically ventilated, has a spontaneous breathing trial been performed? Is the weaning plan defined? Can the patient be extubated today? - Is the ventilation protective? **End-of-ICU plan (ICU discharge plan)** - Have DNR orders, advance directives, or palliative needs been discussed and/or updated? - Is there an ICU discharge plan? Does the patient need rehabilitation? Advanced care planning? **Antibiotics and other drugs** - Are antibiotics indicated? Can broad-spectrum antibiotics be changed to more targeted antibiotics? - Does every drug have a diagnosis or symptom? - Does every patient's diagnosis have all the required medications? - Is there a need for dosing adjustment or therapeutic level monitoring (e.g., renal or hepatic failure)? **Katheter* (Catheter) management** - Can any central lines, drains, or catheters be removed? - Are there signs of central line infection?

* Note: For the last item, the German word for "catheter" was used to fit the acronym.
